# Dissociation and other trauma symptomatology are linked to imbalance in the competing neurobehavioral decision systems

**DOI:** 10.3389/fpsyg.2023.1317088

**Published:** 2024-01-31

**Authors:** Julia C. Basso, Medha K. Satyal, Kevin L. McKee, Sarah Lynn, Daphne Gyamfi, Warren K. Bickel

**Affiliations:** ^1^Department of Human Nutrition, Foods, and Exercise, Virginia Tech, Blacksburg, VA, United States; ^2^Center for Health Behaviors Research, Fralin Biomedical Research Institute at Virginia Tech Carilion, Roanoke, VA, United States; ^3^School of Neuroscience, Virginia Tech, Blacksburg, VA, United States; ^4^Center for Biostatistics and Health Data Science, Virginia Tech, Blacksburg, VA, United States; ^5^Addiction Recovery Research Center, Fralin Biomedical Research Institute at VTC, Roanoke, VA, United States

**Keywords:** depersonalization, derealization, trauma, delay discounting, temporal discounting, competing neurobehavioral decisions systems theory

## Abstract

**Objective:**

Dissociation is a conscious state characterized by alterations in sensation and perception and is thought to arise from traumatic life experiences. Previous research has demonstrated that individuals with high levels of dissociation show impairments in cognitive-emotional processes. Therefore, using the Competing Neurobehavioral Decisions System (CNDS) theory, we used statistical modeling to examine whether dissociative experience and trauma symptoms are independently predicted by impulsivity, risk-seeking, affective state (i.e., anxiety, depression, stress, and negative affect), and trauma history.

**Method:**

In this cross-sectional study design, data were collected via Amazon Mechanical Turk from a total of *n* = 557 English-speaking participants in the United States. Using Qualtrics, participants answered a series of self-reported questionnaires and completed several neurocognitive tasks. Three independent multiple linear regression models were conducted to assess whether impulsivity, risk seeking, affective state, and trauma history predict depersonalization, trauma symptoms, and PTSD symptoms.

**Results:**

As hypothesized, we found that depersonalization and other trauma symptoms are associated with heightened impulsivity, increased risk-seeking, impaired affective states, and a history of traumatic experiences.

**Conclusion:**

We demonstrate that an imbalanced CNDS (i.e., hyperimpulsive/hypoexecutive), as evidenced by decreased future valuation, increased risk seeking, and impaired affective states, predicts heightened depersonalization and other trauma and PTSD symptomatology. This is the first time that dissociation has been connected to delay discounting (i.e., the tendency to place more value on rewards received immediately compared to farther in the future). Interventions that positively impact areas of the CNDS, such as episodic future thinking or mindfulness meditation, may be a target to help decrease dissociative symptoms.

## Introduction

Dissociative disorders, such as dissociative amnesia, depersonalization-derealization disorder, and dissociative identity disorder, are characterized by impairments in the integration of various conscious states including emotion, sensory perception, motor actions, memory, or identity (Brown and Barlow, 2005). Unlike psychotic disorders, such as schizophrenia, individuals with dissociative disorders have an intact sense of reality. Individuals who have dissociative experiences may feel detached from their own bodies, disconnected from their surroundings, or feel as if a known acquaintance is a complete stranger. They may feel as if they are watching their own life from afar, as if they are floating above reality, or as if they are watching a movie of what is actually happening. While the prevalence of clinical dissociative disorders is relatively low, between 1.2 and 2.4%, these experiences commonly occur in individuals with other psychiatric disorders such as depression, anxiety, and post-traumatic stress disorder (PTSD) (Şar, 2014). Additionally, up to 74% of the general population experiences transient symptoms of depersonalization or derealization in their lifetime (Hunter et al., 2004). Both clinical and scientific work indicates that dissociative experiences, especially in clinical disorders, emerge from traumatic experiences (Nijenhuis et al., 1998; Şar, 2014).

Cognitive neuroscience research has shown that dissociative disorders are linked to various cognitive deficits. Specifically, dissociation is associated with emotional deficits, including difficulty in identifying emotional states, blunting of affect, and impaired emotional memory (Medford et al., 2006; Montagne et al., 2007; Simeon et al., 2009). Additionally, individuals with dissociative disorders demonstrate impairments in cognitive processing, especially attentional control and focus (Guralnik et al., 2000, 2007). Functional neuroimaging studies have also found that dissociation is associated with impairments in brain regions necessary for cognitive-emotional processing. For example, compared to healthy controls, when presented with emotionally salient stimuli (e.g., emotional facial expressions), individuals with dissociative disorders show suppressed activity in limbic/paralimbic areas such as the amygdala and insula and heightened activity in executive areas such as the prefrontal cortex (Phillips et al., 2001; Medford et al., 2006; Lemche et al., 2007, 2008). Additionally, new research indicates that dissociation is associated with a slow (~3 Hertz) oscillation in the deep posteriomedial cortex, which functionally decouples activity in this region from activity in other areas of the brain causing a sensation of disconnection between the mind and body (Vesuna et al., 2020).

Considering that dissociation is linked to dysfunction of cognitive-emotional processing, we hypothesized that the Competing Neurobehavioral Decision Systems (CNDS) theory would be a good framework for investigating dissociation. The CNDS theory posits that two systems govern behavior; namely, the reward-driven impulsive system, governed by limbic and paralimbic regions (e.g., nucleus accumbens, amygdala, insula), and the future-driven executive system, governed by prefrontal and parietal regions (e.g., dorsolateral prefrontal cortex) (McClure and Bickel, 2014; Bickel et al., 2018). In balance, these systems support a wide range of healthy behaviors; however, when they are imbalanced, maladaptive and pathological behaviors result such as those exhibited in substance use disorders, obesity, and Type 2 diabetes (e.g., excessive substance use, overeating, and sedentary behavior) (Bickel et al., 2014a, 2019). We hypothesize that a heightened CNDS imbalance (e.g., hyperfunctioning of impulsive areas and hypofunctioning of executive areas) significantly correlates to heightened dissociative experiences and other trauma symptoms (e.g., depression, anxiety, sleep issues, and sexual problems). Here, we examined CNDS balance via a compilation of self-reported measures and neurocognitive assessments that have been previously mapped to CNDS brain regions via functional magnetic resonance imaging or other neuroimaging techniques. First, we utilized the neuroeconomic task of temporal discounting (TD, also known as delay discounting), which provides a behavioral measure [i.e., ln (k)] that identifies the relative balance between the impulsive and executive systems and has traditionally been used to assess CNDS balance (Bickel et al., 2018). TD has been identified as a trans-disease process across many neuropsychiatric disorders including major depressive disorder, schizophrenia, borderline personality disorder, bipolar disorder, bulimia nervosa, and binge eating disorder (Bickel et al., 2012, 2019; Amlung et al., 2019); however, to our knowledge, TD has never been looked at in relation to dissociation. Other measures to evaluate CNDS balance include probability discounting and affective state measures, all of which have been mapped to regions of the CNDS (e.g., prefrontal cortex, amygdala, nucleus accumbens) (Gray et al., 2002; Perlstein et al., 2002; Beer et al., 2010; Funahashi and Andreau, 2013; Yang et al., 2016; Liu et al., 2017; Hare and Duman, 2020).

The present study used three independent linear regression models to examine whether dissociative experience and trauma symptoms as measured through the Trauma Symptoms Checklist and PTSD Symptom Scale are predicted by impulsivity, risk-seeking, affective state, and trauma history. We hypothesized that all three of our primary outcomes would be significantly predicted independently by increased impulsivity, risk-seeking, negative affective state, and traumatic experiences. We present the resulting models and a discussion regarding the prominent neurobehaviors associated with heightened dissociation and the clinical implications of such findings.

## Methods

### Recruitment

Data were obtained on Amazon Mechanical Turk (mTurk), an online crowdsourcing data collection platform that allows users to complete Human Intelligence Tasks (HITs) for compensation. The HIT was available to mTurk workers with a high (>90%) HIT approval rate located in the United States. Participants who indicated English as their primary language were eligible to complete the study. Additionally, in order to screen out machine input, participants needed to pass a CAPTCHA test and correctly answer a series of three text-entry attention check questions that were evaluated for accuracy. Participants with and without a history of trauma were included in the study. All screening and demographic questions, self-report measures, and discounting tasks were programmed and presented in Qualtrics. Self-report questionnaires and cognitive tasks were presented in a randomized order following screening and demographic questions. Participants were compensated for completing the questionnaire and received a bonus compensation if their data passed attention checks. All study procedures were approved by the Virginia Tech Institutional Review Board.

### Participants

A total of *N* = 802 completed the HIT. To be included in the analysis, participants needed to fully complete the questionnaire (i.e., progress = 100%). If a participant completed the survey multiple times, only the first entry was included. Because age was a covariate in the majority of the analyzes, one participant who did not provide an appropriate response for age was excluded. Final data analysis was completed on a total of *n* = 557 participants.

### Measures

Ten validated self-reported questionnaires and neuropsychological assessments were administered to all participants. For our dependent variables, to assess the dissociative experience, we utilized the Cambridge Depersonalization Scale, and to assess trauma symptomatology, we used the Trauma Symptoms Checklist and the Post-Traumatic Stress Disorder Symptom Scale. Regarding our independent variables of interest, we assessed three aspects of CNDS balance: temporal discounting via the Delay Discounting Task; risk aversiveness via the Probability Discounting Task; and affective state via the Beck Depression Inventory, Beck Anxiety Inventory, Positive and Negative Affect Schedule, and Perceived Stress Scale. Additionally, to assess trauma history, we utilized the Trauma History Questionnaire.

### Demographics

Demographic information was collected to include as covariates in data analysis as previous reports have shown that our outcomes of interest, including temporal discounting, stress, depression, and anxiety may be impacted by these demographic characteristics (Miech and Shanahan, 2000; Zimmerman and Katon, 2005; Altemus et al., 2014; Bickel et al., 2014b). Self-reported demographic information including age, sex, race, ethnicity, household income, education, and employment status was collected from all participants who were eligible for the study based on their responses to screening questions. If reported personal income exceeded household income, then the value for household income was replaced with the value for personal income. Household income was then categorized based on low (<$40,000 per year), middle ($40,000 to $125,000 per year), and high (>$125,000 per year) strata (Semega et al., 2017).

### Assessment of dissociation

#### Cambridge Depersonalization Scale

The Cambridge Depersonalization Scale (CDS) is a reliable (Cronbach’s alpha = 0.89) questionnaire that assesses dissociative symptoms (Sierra and Berrios, 1996, 2000). The CDS was validated against the DSM-IV criteria for depersonalization disorder as well as the depersonalization subscale of the Dissociative Experiences Scale. The CDS consists of 29 items; each item is scored on a 5-point scale for frequency and a 6-point scale for duration. Items are summed for a total score as well as six subscale scores including frequency, duration, anomalous body experience, emotional numbing, anomalous subjective recall, and alienation from surroundings. The full questionnaire is available in the original publication (Sierra and Berrios, 2000).

### Assessment of trauma symptoms

#### Trauma Symptoms Checklist-40

The Trauma Symptoms Checklist-40 (TSC) is a valid and highly reliable (Cronbach’s alpha =0.90) questionnaire that assesses distress arising from past trauma (Elliott and Briere, 1992). The TSC-40 consists of 40 items scored on a 4-point Likert scale. Items are summed for a total score and six subscale scores including anxiety, depression, dissociation, sexual abuse trauma index (SATI), sexual problems, and sleep disturbance.

#### Post-Traumatic Stress Disorder Symptom Scale

The self-report version of the Post-Traumatic Stress Disorder (PTSD) Symptom Scale is a valid, internally consistent (Cronbach’s alpha =0.91), and reliable (test–retest r = 0.74) questionnaire that assesses the severity of PTSD symptoms (Foa et al., 1993). It consists of 17 items scored on a 4-point Likert scale. Items are summed for a total score with a range of possible scores from 0 to 51.

### Assessment of CNDS balance

#### Delay discounting task

The five-trial adjusting delay discounting task assesses temporal discounting, a measure of future valuation that assesses the indifference point at which an individual is willing to wait to receive a larger, later reward versus a smaller, more immediate reward (Koffarnus and Bickel, 2014). Participants were asked to choose between an immediate $500 reward or delayed $1,000 reward at different time delays, with the delay increasing or decreasing in the subsequent trial based on the prior response with a total of five trials. The ED_50_, the delay at which the value of the larger reward is reduced by 50%, is provided by the indifference point in the task. The delay discount rate (k), the rate at which the value of $1,000 diminishes as a function of time delay, is calculated as the inverse of the ED_50_ (Yoon and Higgins, 2008; Koffarnus and Bickel, 2014). The natural log-transformed delay discount rate (lnk) is used in all analyzes, with higher values of lnk indicating greater impulsivity.

#### Probability discounting task

Probability discounting is a distinct measure from delay discounting, with probability discounting measuring an individual’s level of risk aversiveness (Shead and Hodgins, 2009; McKerchar and Renda, 2012; Green et al., 2014a,b; Hart et al., 2019). Participants were asked to choose between a certain $50 reward or a probabilistic $100 reward. The probability of the larger reward increased or decreased in the subsequent trial based on the prior response for a total of five trials (Cox and Dallery, 2016). The probability discount rate (h), the rate at which the value of $100 diminishes as a function of odds against, is calculated similarly to k described above. The natural log-transformed probability discount rate (lnh) is used in all analyzes, with higher values of lnh indicating greater risk aversiveness.

#### Beck Depression Inventory

The Beck Depression Inventory (BDI-II) is a validated and widely used questionnaire that assesses symptoms of depression (Beck et al., 1996; Dozois et al., 1998). The BDI-II consists of 21 items scored on a 4-point Likert scale. Items are summed for a total score with a range of scores from 0 to 63. The BDI-II has high internal consistency (Cronbach’s alpha = 0.92–0.93).

#### Beck Anxiety Inventory

The Beck Anxiety Inventory (BAI) is a valid and reliable questionnaire that assesses symptoms of anxiety (Beck et al., 1988). The BAI has high internal consistency (Cronbach’s alpha = 0.92) and test–retest reliability (*r* = 0.75). The BAI consists of 21 items scored on a 4-point Likert scale. Items are summed for a total score with a range of scores from 0 to 63.

#### Positive and negative affect schedule – Short Form

The Positive and Negative Affect Schedule – Short Form (PANAS – SF) is a validated and reliable questionnaire that assesses both positive (PA) and negative affect (NA) (Watson et al., 1988). The PANAS consists of two 10-item mood scales scored on a 5-point Likert scale. Items are summed for a positive affect score and a negative affect score, with each score ranging from 10 to 50. For this study, we only evaluated the negative affect subscale.

#### Perceived stress scale

The Perceived Stress Scale (PSS) is a valid, internally consistent (Cronbach’s alpha = 0.84–0.86), and reliable (test–retest r = 0.85) questionnaire that assesses psychological stress (Cohen et al., 1983). The PSS consists of 10 items scored on a 5-point Likert scale. Items are summed for a total score with potential scores from 0 to 40.

### Assessment of trauma history

#### Trauma history questionnaire

The Trauma History Questionnaire (THQ) is a valid and reliable (test–retest r = 0.70) questionnaire that assesses past experience with potentially traumatic events (Green, 1996; Hooper et al., 2011). The THQ consists of 24 yes/no items regarding past traumatic experiences (e.g., natural disaster, serious accident, or unwanted sexual contact). The number of items endorsed are summed for a total event score. The full questionnaire is available in Hooper et al. (2011).

### Statistics

Three independent multiple linear regression models were conducted to assess whether impulsivity, risk seeking, affective state, and trauma history predict depersonalization, trauma symptoms, and PTSD symptoms. Additionally, three linear regression models were conducted to assess the ability of impulsivity, risk seeking, affective state, and trauma history to predict depersonalization, trauma symptoms, and PTSD symptoms after controlling for demographic variables (i.e., age, race, ethnicity, education, income, employment, sex).

All of our affective state measures were found to have significant collinearity. Therefore, to obtain one factor that encapsulated negative affective state, a principal components analysis (PCA) was run on the outcomes of 4 affective state questionnaires: BAI, BDI, PSS, and the negative affect subscale of the PANAS. The suitability of PCA was assessed prior to analysis. Inspection of the correlation matrix showed that all variables had at least one correlation coefficient greater than 0.3. The overall Kaiser-Meyer-Olkin (KMO) measure was 0.76, classifications of ‘middling’ to ‘meritorious’ (Kaiser, 1974). Bartlett’s test of sphericity was statistically significant (*p* < 0.001), indicating that the data was likely factorizable. PCA revealed one component that had eigenvalues greater than one and which explained 74.8% of the total variance. Visual inspection of the scree plot indicated that this one component should be retained (Cattell, 1966).

To determine statistical significance, an alpha value of 0.05 was utilized. IBM SPSS Statistics Version 27 was utilized for all analyzes.

## Results

### Demographics

Table 1 summarizes demographic characteristics of the sample participants. The sample was primarily male (57.6%), White (77.6%), non-Hispanic (82.9%), college educated (53.7%), employed full-time (78.8%), and from a middle-income household (63.0%).

**Table 1:** Demographic characteristics of study sample.

**Race**		**Employment status**	
%White/Caucasian	77.6	%Working full time	78.8
%Black/African American	15.4	%Working part time	11.5
%Asian	3.9	%Homemaker	2.2
%Other	3.1	%Not working	7.5
**Ethnicity**		**Sex**	
%Hispanic	17.1	%Female	42.4
%Non-Hispanic	82.9	%Male	57.6
**Education**		**Household income**	
%High school/GED or lower	8.6	%Low income	27.8
%Some college	27.6	%Middle income	63.0
%College degree	53.7	%High income	9.2
%Advanced degree	10.1	**Age**	35.23 (0.44)

“Other” race includes those who answered American Indian/Alaska Native, Pacific Islander, or Other. “Not working” includes those who answered not working, laid off, retired, or disability.

### Depersonalization

The unadjusted regression model was significant (*F* (4, 552) = 189.036, *p* < 0.001) and explained 57.5% of the variance in the outcome. The model indicated that impulsivity (t = 5.461, *p* < 0.001), risk-seeking (*t* = −5.580, *p* < 0.001), and affective state (*t* = 22.103, *p* < 0.001) were significant predictors of depersonalization while trauma history (*t* = −0.282, *p* = 0.778) was not a significant predictor (Table 2).

**Table 2:** Multiple linear regression models predicting depersonalization.

Dependent variable: Cambridge Depersonalization Scale score	R	R^2^	Adjusted R^2^	F	Beta	*t*	*p*
Unadjusted**	0.760	0.578	0.575	189.036			<0.001
Impulsivity**					0.163	5.461	<0.001
Risk-seeking**					−0.166	−5.850	<0.001
Affective State**					0.667	22.103	<0.001
Trauma History					−0.008	−0.282	0.778
Adjusted**	0.778	0.606	0.598	76.105			<0.001
Impulsivity**					0.136	4.575	<0.001
Risk-seeking**					−0.140	−4.931	<0.001
Affective State**					0.615	19.387	<0.001
Trauma History					0.021	0.736	0.462
Age					−0.035	−1.200	0.231
Race					0.018	0.634	0.527
Ethnicity					−0.079	−2.653	0.527
Income					0.044	1.538	0.125
Education**					0.111	3.676	<0.001
Employment					−0.031	−1.063	0.288
Sex					−0.003	−0.120	0.904

^*^p < 0.05; ^**^p < 0.01.

The adjusted regression model was significant (*F* (11, 545) = 76.105, *p* < 0.001) and explained 59.8% of the variance in the outcome. The model indicated that impulsivity (*t* = 4.575, *p* < 0.001), risk-seeking (t = −4.931, *p* < 0.001), and affective state (*t* = 19.385, *p* < 0.001) were significant predictors of depersonalization after controlling for age, race, ethnicity, education, income, employment, and sex (Table 2).

### Trauma symptoms

The unadjusted regression model was significant (*F* (4, 552) = 501.313, *p* < 0.001) and explained 78.3% of the variance in the outcome. The model indicated that impulsivity (*t* = 5.123, *p* < 0.001), risk seeking (*t* = −3.665, *p* < 0.001), affective state (t = 38.409, *p* < 0.001), and trauma history (*t* = 2.123, *p* = 0.034) were significant predictors of trauma symptoms (Table 3).

**Table 3:** Multiple linear regression models predicting trauma symptoms.

Dependent variable: Trauma symptoms checklist score	R	R^2^	Adjusted R^2^	F	Beta	*t*	*p*
Unadjusted**	0.886	0.784	0.783	501.313			<0.001
Impulsivity**					0.109	5.123	<0.001
Risk-seeking**					−0.074	−3.665	<0.001
Affective State**					0.829	38.409	<0.001
Trauma History*					0.044	2.123	0.034
Adjusted**	0.899	0.808	0.804	208.547			<0.001
Impulsivity**					0.085	4.121	<0.001
Risk-seeking*					−0.049	−2.482	0.013
Affective State**					0.790	35.668	<0.001
Trauma History**					0.070	3.445	0.001
Age					−0.001	−0.042	0.967
Race*					−0.042	−2.143	0.033
Ethnicity**					−0.101	−4.853	<0.001
Income**					0.053	2.686	0.007
Education**					0.081	3.813	<0.001
Employment					−0.037	−1.837	0.067
Sex					−0.006	−0.302	0.763

*p < 0.05; **p < 0.01.

The adjusted regression model was significant (*F* (11, 545) = 208.547, *p* < 0.001) and explained 80.4% of the variance in the outcome. The model indicated that impulsivity (*t* = 4.121, *p* < 0.001), risk seeking (*t* = −2.482, *p* = 0.013), affective state (*t* = 35.668, *p* < 0.001), and trauma history (*t* = 3.445, *p* = 0.001) were significant predictors of trauma symptoms after controlling for age, race, ethnicity, education, income, employment, and sex (Table 3).

### PTSD symptoms

The unadjusted regression model was significant (*F* (4, 552) = 157.895, *p* < 0.001) and explained 53.0% of the variance in the outcome. The model indicated that risk-seeking (t = −3.233, *p* = 0.001), affective state (*t* = 19.922, *p* < 0.001), and trauma history (*t* = 7.500, *p* < 0.001) were significant predictors of PTSD symptoms while impulsivity (*t* = 0.551, *p* = 0.582) was not a significant predictor (Table 4).

**Table 4:** Multiple linear regression models predicting PTSD symptoms.

Dependent variable: PTSD symptoms scale score	R	R^2^	Adjusted R^2^	F	Beta	*t*	*p*
Unadjusted**	0.730	0.534	0.530	157.895			<0.001
Impulsivity					0.017	0.551	0.582
Risk-seeking**					−0.097	−3.233	0.001
Affective state**					0.632	19.922	<0.001
Trauma history**					0.227	7.500	<0.001
Adjusted**	0.742	0.550	0.541	60.592			<0.001
Impulsivity					0.001	0.031	0.975
Risk-seeking**					−0.084	−2.786	0.006
Affective State**					0.594	17.539	<0.001
Trauma History**					0.249	8.056	<0.001
Age					−0.021	−0.681	0.496
Race					−0.037	−1.239	0.216
Ethnicity*					−0.076	−2.387	0.017
Income					0.018	0.607	0.544
Education*					0.071	2.185	0.029
Employment					−0.044	−1.424	0.155
Sex					−0.057	−1.884	0.060

^*^p < 0.05; ^**^p < 0.01.

The adjusted regression model was significant (*F* (11,545) = 60.592, *p* < 0.001) and explained 54.1% of the variance in the outcome. The model indicated that risk-seeking (*t* = −2.786, *p* = 0.006), affective state (*t* = 17.539, *p* < 0.001), and trauma history (*t* = 8.056, *p* < 0.001) were significant predictors of PTSD symptoms after adjusting for age, race, ethnicity, education, income, employment, and sex (Table 4).

## Discussion

Traumatic experiences can produce psychological states characterized by depression, anxiety, sleep disturbances, sexual problems, and dissociation, a conscious state characterized by a disconnect from thoughts, feelings, emotions, memories, or sense of self. In this population-based research study, we utilized linear regression to examine depersonalization and other trauma symptomatology from the perspective of the CNDS theory, which posits that neurobehaviors are governed by two major neuroanatomical circuits, namely the impulsive and executive systems. Our findings support our overall hypothesis, showing that depersonalization and trauma symptoms are associated with heightened impulsivity, increased risk-seeking, impaired affective states, and a history of traumatic experiences. These results suggest that trauma, dissociation, and other psychological states are associated with a dysregulated CNDS, geared towards a hyperactive–impulsive system and a hypoactive executive system.

### Depersonalization, trauma, and PTSD symptoms are predicted by CNDS imbalance

The present study found that greater depersonalization and trauma symptomatology is predicted by CNDS imbalance as indicated by heightened impulsivity, risk-seeking, and negative affect. Additionally, PTSD symptomatology is predicted by CNDS imbalance as indicated by heightened risk-seeking and negative affect. This is the first time that the psychological state of dissociation has been linked to impulsivity as measured by temporal discounting (TD). In line with our findings, previous research has shown that TD is a transdiagnostic process across a range of other neuropsychiatric disorders including major depressive disorder, schizophrenia, borderline personality disorder, and bipolar disorder (Bickel et al., 2012, 2019; Amlung et al., 2019). Interestingly, dissociation is a common symptom in many of these mental health disorders and is one of the factors assessed in our measure of trauma symptomatology (Spiegel et al., 1996; Nijenhuis et al., 1998; Stiglmayr et al., 2008; Stein et al., 2013). Regarding the fact that impulsivity is a predictor of trauma symptomatology as measured by the TSC but not the PTSD Symptom Scale, this discrepancy may be due to the nature of these self-report scales. The TSC is a list of 40 experiences that may have occurred over the past 2 months including headaches, stomach problems, anxiety attacks, uncontrollable crying, feelings of guilt, and sexual problems. The PTSD Symptom Scale asks 20 detailed questions such as how often individuals try not to think or talk about the traumatic event, feel distant or cut off from the people around them, or feel as if their future hopes or plans will not come true. That is, these questionnaires are qualitatively different and though there is overlap between the two in terms of symptoms, the TSC includes more somatic- (body) and sexual-based questions. Future studies should determine if impulsivity is better predicted by somatic rather than cognitive trauma symptomatology.

In regard to risk-seeking, previous research has shown that individuals with dissociative disorders engage in a variety of risky behaviors, including alcohol and substance abuse, self-mutilation/harm, and unsafe sexual practices (Saxe et al., 2002; Zurbriggen and Freyd, 2004; Foote et al., 2008; Kianpoor and Bakhshani, 2012). Hart et al. (2019) found that individuals with bipolar affective disorder or schizophrenia demonstrate heightened rates of probability discounting (i.e., risk aversiveness) compared to healthy controls, indicating that different mental health disorders may be associated with different levels of risk-taking behavior. Heightened rates of TD and lower rates of probability discounting may be a good behavioral marker of dissociative disorders.

As limited research has investigated the relationship between dissociation and affective state in non-clinical populations, our findings represent some of the first data to show the dynamic relationship between dissociation and other mood states. Depersonalization is predicted by increased negative affect, suggesting that individuals with high depersonalization experience more negative emotional states. This finding supports previous suggestions that dissociation occurs due to emotional overstimulation; that is, when emotions become too extreme, the individual dissociates to escape the experience (Mosquera et al., 2014). In clinical dissociative disorders, dissociation is considered a pathological state. However, in nonclinical populations, dissociation may be a neurobiological adaptation serving as a protective mechanism to guard against present or future negative emotions (Briere, 2006; Oathes and Ray, 2008; Schimmenti and Caretti, 2016). Though dissociation often co-occurs with other neuropsychiatric disorders, such as depression and anxiety, research has shown that they are in fact distinct disorders (Lipsanen et al., 2004). Because of this close link, the treatment of neuropsychiatric disorders may benefit from addressing co-occurring dissociative symptomatology (Prasko et al., 2016).

### Trauma and PTSD symptoms are predicted by trauma history

We also found that trauma history predicted current trauma and PTSD symptoms; individuals who experienced more traumatic events showed heightened levels of trauma and PTSD symptomatology. As the lasting psychological impacts of traumatic experiences are well-documented in the literature (Breslau et al., 1998; Kazantzis et al., 2010; De Venter et al., 2013), this finding is in line with this body of work. Additionally, others have reported that past traumatic experiences are associated with heightened TD, indicating that these factors often co-occur (Simmen-Janevska et al., 2015; van den Berk-Clark et al., 2018). While previous findings show that dissociation occurs as a result of traumatic experiences (Nijenhuis et al., 1998; Şar, 2014), a model known as the Trauma Model (Dalenberg et al., 2012), trauma history was not a significant predictor of depersonalization in the present study. This may be due to the type of self-reported questionnaire that was used rather than in-depth interviews, which are often used to capture information about trauma history (Center for Substance Abuse Treatment (US), 2014). Alternatively, the Taxon Model suggests that depersonalization occurs on a spectrum from normal to pathological dissociation (Waller et al., 1996). The latter is associated with highly traumatized individuals (approximately 3.5% of the general population) who experience a cluster of symptoms associated with severe dissociative psychopathology (e.g., amensia for recent episodic memories, identity alteration). The fact that we did not see a relationship between trauma history and depersonalization suggests that the majority of our population may be those within the normal range of depersonalization. In fact, the highest frequency of participants reported having zero depersonalization symptoms, with 65.5% of the population reporting a score that is not consistent with depersonalization disorder (i.e., a total score of less than 113 on the Cambridge Depersonalization Scale).

The psychological impacts of traumatic experiences may be due to alterations in brain connectivity, particularly in the frontoparietal executive network, which persist even decades after the traumatic event (Yu et al., 2019). Additionally, Lebois et al. (2021) found that functional network connections can be used to predict severe dissociation in PTSD, with the frontoparietal executive network being one of the most important predictors. As the frontoparietal network is also involved in TD (Clewett et al., 2014), our present findings suggest that future work using neuroimaging methods is warranted to elucidate the relationship between dissociation, trauma, and CNDS function.

### Limitations and future directions

Though this population study provides novel insights into dissociation, it is not without its limitations. First, we collected our data using Amazon’s mTurk crowdsourcing tool, which allowed for a large sample size. To optimize data quality, participants needed to pass a CAPTCHA response and answer several attention-check questions (Hunt and Scheetz, 2019). This technique helped to minimize the known data quality limitations of mTurk. Though the demographics of mTurk Workers are not always generalizable to the general public, the sample collected is more diverse than a college campus or any single geographic location. Additionally, recent research has shown that an mTurk sample of individuals screened for PTSD showed comparable characteristics to undergraduate, community, and treatment-seeking samples, indicating that the results are applicable to the general public (Engle et al., 2020). Our laboratory results also show consistent results between mTurk and in-lab samples. Further, we did not exclude based on current or past history of neuropsychiatric diagnosis. However, these inclusive criteria provided for a more expansive range of dissociative and trauma symptomatology in our dataset. Additionally, although we utilized a validated questionnaire to assess trauma history, there are known methodological challenges with self-report of traumatic events related to memory accuracy, recall, and reporting (Corcoran et al., 2000; Bardeen and Benfer, 2019).

Future studies should examine the various neurobehaviors examined here in individuals with a primary diagnosis of dissociative disorders or PTSD as well as examine the role of dissociative symptoms and delay discounting in individuals with other neuropsychiatric disorders. Considering that some of the neurobehaviors explored here (e.g., delay discounting, affective state) are plastic, future studies should consider using an interventional approach to modulate these neurobehaviors and examine the effect on dissociative state and trauma symptomatology. Finally, future research using a neuroimaging or neurophysiological approach is needed to investigate the neural mechanisms underlying the relationships between dissociation, trauma, and CNDS function.

## Conclusion

Here we demonstrate that an imbalanced CNDS (i.e., hyperimpulsive/hypoexecutive), as evidenced by decreased future valuation, increased risk seeking, and impaired affective states, predicts heightened depersonalization and other trauma and PTSD symptomatology. This is the first time that dissociation has been connected to temporal discounting. Additionally, and as hypothesized, we found that trauma history predicted trauma and PTSD symptomatology; however, depersonalization was not associated with trauma history. These data suggest that individuals with heightened levels of depersonalization and other trauma symptoms focus on a shorter temporal frame or window, placing heightened value on brief, immediate reinforcers over longer, delayed reinforcers (Bickel et al., 2014a; Bickel and Athamneh, 2020). This may be because the dissociative and trauma symptoms experienced force individuals to remain in a more present-based state. These individuals may put less value on a future fraught with dissociative symptomatology, anxiety, stress, or other negative affective states. Interventions that enhance future thinking and decrease delay discounting may be clinically helpful for individuals with depersonalization and other trauma symptomatology.

## Data availability statement

The raw data supporting the conclusions of this article will be made available by the authors, without undue reservation.

## Ethics statement

The studies involving humans were approved by the Virginia Tech Institutional Review Board. The studies were conducted in accordance with the local legislation and institutional requirements. The participants provided their written informed consent to participate in this study.

## Author contributions

JB: Conceptualization, Data curation, Formal analysis, Methodology, Project administration, Validation, Visualization, Writing – original draft, Writing – review & editing. MS: Formal analysis, Writing – original draft, Writing – review & editing. KM: Formal analysis, Writing – original draft, Writing – review & editing. SL: Writing – review & editing. DG: Writing – review & editing. WB: Funding acquisition, Resources, Supervision, Writing – review & editing.
